# GelFAP: Gene Functional Analysis Platform for *Gastrodia elata*

**DOI:** 10.3389/fpls.2020.563237

**Published:** 2020-10-22

**Authors:** Jiaotong Yang, Qiaoqiao Xiao, Jiao Xu, Lingling Da, Lanping Guo, Luqi Huang, Yue Liu, Wenying Xu, Zhen Su, Shiping Yang, Qi Pan, Weike Jiang, Tao Zhou

**Affiliations:** ^1^Source Institute for Chinese and Ethnic Materia Medica, Guizhou University of Traditional Chinese Medicine, Guiyang, China; ^2^College of Biological Sciences, China Agricultural University, Beijing, China; ^3^National Resource Center for Chinese Materia Medica, China Academy of Chinese Medical Sciences, Beijing, China; ^4^College of Horticulture, Qingdao Agricultural University, Qingdao, China

**Keywords:** *Gastrodia elata*, co-expression network, functional module, gene functional analysis platform, functional enrichment analysis

## Abstract

*Gastrodia elata*, also named Tianma, is a valuable traditional Chinese herbal medicine. It has numerous important pharmacological roles such as in sedation and lowering blood pressure and as anticonvulsant and anti-aging, and it also has effects on the immune and cardiovascular systems. The whole genome sequencing of *G. elata* has been completed in recent years, which provides a strong support for the construction of the *G. elata* gene functional analysis platform. Therefore, in our research, we collected and processed 39 transcriptome data of *G. elata* and constructed the *G. elata* gene co-expression networks, then we identified functional modules by the weighted correlation network analysis (WGCNA) package. Furthermore, gene families of *G. elata* were identified by tools including HMMER, iTAK, PfamScan, and InParanoid. Finally, we constructed a gene functional analysis platform for *G. elata*^1^. In our platform, we introduced functional analysis tools such as BLAST, gene set enrichment analysis (GSEA), and *cis-*elements (motif) enrichment analysis tool. In addition, we analyzed the co-expression relationship of genes which might participate in the biosynthesis of gastrodin and predicted 19 mannose-binding lectin antifungal proteins of *G. elata*. We also introduced the usage of the *G. elata* gene function analysis platform (GelFAP) by analyzing *CYP51G1* and *GFAP4* genes. Our platform GelFAP may help researchers to explore the gene function of *G. elata* and make novel discoveries about key genes involved in the biological processes of gastrodin.

## Introduction

*Gastrodia elata*, a kind of perennial herb of Orchidaceae, is one of the traditional Chinese herbal medicines. The growth cycle of *G. elata* is generally about 3 years, including the development stages of the seed, protocorm, juvenile tuber, immature tuber, mature tuber, and scape (Yuan et al., 2018). *G. elata* is a typical heterotrophic plant, which has a symbiotic relationship with at least two fungi during its life cycle. One is *Mycena* that offers nutrition for the seed germination of *G. elata*, and the other is *Armillaria mellea* that offers nutrition and energy for the vegetative propagation corms of *G. elata* development into tubers (Xu, 1981, 1989). The mannose-binding lectin antifungal proteins of *G. elata* (GAFPs) play important roles in its growth during *G. elata* and *A. mellea*, establishing a stable symbiotic association (Yuan et al., 2018). *G. elata* has important functions such as in sedation and lowering blood pressure and as anticonvulsant and anti-aging, and it also has effects on the immune and cardiovascular systems. Its pharmacological action makes it widely used in clinical settings (Shan et al., 2016). As an important medicinal plant, *G. elata* has many active chemical ingredients, such as gastrodins, 4-hydroxybenzyl alcohols, vanillyl alcohols, vanillins, polysaccharides, sterols, and organic acids (Shan et al., 2016). Among them, gastrodin is one of the important components for its beneficial effects. Gastrodin biosynthesis pathway from toluene to 4-hydroxytoluene can be catalyzed by monooxygenase of cytochrome P450 (CYP450) (Carmona et al., 2009), and then CYP450 further catalyzes the oxidation of 4-hydroxytoluene to p-hydroxybenzyl alcohol; finally, glycogenase is synthesized through glycosyltransferase (UGT) (Tsai et al., 2016). Therefore, exploring the function of genes that can catalyze the synthesis of gastrodin from the CYP450 and UGT gene family will help to explore the molecular mechanism of gastrodin biosynthesis.

The development of high-throughput sequencing technology has greatly enriched the research methods in the field of life sciences, and it not only improves the efficiency of scientific research but also promotes the development of basic research. In the past decade, whole genome sequencing had been completed in typical model plants and crops, and many species even owned their gene function analysis platforms, which were established by the integration of multiple omics data. Reiser et al. (2017) had established the Arabidopsis Information Resource (TAIR) platform, which covered detailed functional annotation information of each gene and various auxiliary analysis tools, thereby greatly improving research efficiency in scientific fields. Tian et al. (2018) had also built a gene function analysis platform MCENet, which contained a large number of *Zea mays* gene co-expression networks constructed by transcriptomic data and gene function analysis tools, so as to study gene function and synergy between different genes. Recently, Wang et al. (2020) analyzed the genomics data of 13 species in 9 genera of Malvaceae, such as genome-wide association analysis site (GWAS) information and single nucleotide mutation site (SNP) information, as well as a total of 374 sets of transcriptomic and proteomic data, and established a functional genomic hub for Malvaceae plants, which provided a powerful online analysis tool for scientists to carry out mallow family gene function analysis. Therefore, it is necessary to develop a gene function analysis platform for *G. elata* by integrating various annotations, which may contribute to deeper gene function analysis and mining.

The whole genome sequencing of *G. elata* was completed in 2018 (Yuan et al., 2018), making a certain accumulation in transcriptome data of *G. elata*. We collected the transcriptome data of 39 samples, and of these samples, 27 were from the Sequence Read Archive (SRA) in the National Center for Biotechnology Information (NCBI) and 12 were generated by our group. In order to use these data adequately and effectively, we constructed the co-expression network of *G. elata* and identified its functional modules to predict gene function. Furthermore, we constructed a *G. elata* gene function analysis platform (GelFAP) with analysis tools, such as BLAST, GSEA, and *cis-*element enrichment analysis tools, which will help to further explore the novel functions of genes in *G. elata*.

## Materials and Methods

### RNA-Seq Data Processing

The quality control of *G. elata* transcriptome data was performed by FastQC software (version 0.11.2). After removing the unqualified transcriptome data samples, we used TopHat (version 2.1.0) (Trapnell et al., 2009) to map the clean reads to the reference genome and calculated the fragments per kilobase of exon model per million reads mapped (FPKM) values by the Cufflinks software (version 2.2.1) (Trapnell et al., 2010).

### Co-expression Network Construction

Here, the Pearson correlation coefficient (PCC) algorithm was used to construct the gene co-expression networks of *G. elata*. We firstly calculated the correlation between different genes according to the expression values of genes in all 37 samples. Genes with high correlation had similar expression patterns in different samples, which could be considered as gene pairs with co-expression relationship. Then, we calculated the network density and the scale-free topology fitting index *R*^2^ based on the PCC changes and selected the appropriate PCC to construct the gene co-expression network based on the maximizing scale-free topology fitting index *R*^2^ and relative small network density. Correlation can be evaluated by PCC, and the formula is as follows:

$${\text{PCC}}_{xy}=\frac{\sum_{i=1}^{n}\left(x_{i}-\bar{x}\right)\left(y_{i}-\bar{y}\right)}{\sqrt{\sum_{i=1}^{n}{\left(x_{i}-\bar{x}\right)}^{2}\cdot \sum_{i=1}^{n}{\left(y_{i}-\bar{y}\right)}^{2}}}$$

PCC*_*xy*_* is the Pearson correlation coefficient between gene *x* and gene *y*, *n* represents the total number of samples, *x*_*i*_ represents the FPKM values of gene *x* in the *i* sample, *y*_*i*_ represents the FPKM value of gene *y* in sample *i*, $\bar{x}$ represents the average value of gene *x* in *n* samples, and $\bar{y}$ is the average value of gene *y* in *n* samples.

### Gene Set Enrichment Analysis

Gene set enrichment analysis was used as a method for annotating gene sets by calculating the degree of overlap between a specific gene set and various clearly defined gene sets and then defining an enriched gene set by the hypergeometric test, Fisher’s exact test, or *χ^2^* test. Multiple test correction methods for GSEA, including Yekutieli, Bonferroni, Hochberg, Hochberg, Hommel, and Holm, could be used to reduce the false positive rate of GSEA analysis. These methods could perform enrichment analysis on gene ontology (GO) annotations, Kyoto Encyclopedia of Genes and Genomes (KEGG) annotations, and Pfam domain of specific gene sets (Yi et al., 2013). The hypergeometric test was set as a default method for users to perform gene set enrichment analysis. The formula is as follows:

$$P=\frac{\left(\begin{array}{c} n\\ k \end{array}\right)\left(\begin{array}{c} N-n\\ K-k \end{array}\right)}{\left(\begin{array}{c} N\\ K \end{array}\right)}$$

*N* represents the number of genes in *G. elata*, *K* represents the number of genes in an annotated gene set a, *n* represents the number of genes submitted by the user, and *k* represents the overlapped number of genes submitted by the user and the same genes in gene set a.

### Enrichment Analysis of *Cis-*Elements (Motifs)

For the genes which needed to be analyzed, we used the following steps to calculate the *Z* score and *P* value of each motif. Firstly, we scanned the promoter region (1k, 2k, or 3k from annotated genes based on the gene structure “gff” file) of each gene that was submitted by the user and obtained the number of matches for each motif. Secondly, we selected genes to form a gene list from *G. elata* genome for 1,000 times randomly, and the number of genes was equal to the number of users who have submitted. Thirdly, we scanned the 3-kb promoter region of each gene list and calculated the average number of each motif. Finally, we calculated the *Z* score and *P* value of each motif based on the following formula. If the *P* value was less than 0.05, it meant that the motif was significantly enriched.

$$Z=\frac{\bar{X}-\mu }{\sigma /\sqrt{n}}$$

$$P\text{value}=1-P\text{norm}\left(\bar{X},\mu ,\frac{\sigma }{\sqrt{n}}\right)$$

### Module Identification and Annotation

We used the weighted gene correlation network analysis (WGCNA) package (Langfelder and Horvath, 2008) of R language to identify the functional modules. The process mainly included four steps. Firstly, we defined the gene co-expression correlated matrix, which weighted the correlation between genes, and determined the software threshold *β* based on the maximizing scale-free topology fitting index (*R*^2^). Secondly, the blockwiseModules function was used to construct a scale-free network, and then module partition analysis was executed to identify functional modules. Thirdly, modules were defined by the dynamic tree cutting algorithm. Lastly, modules with high similarity were merged to get the final modules. Through this package, we identified the functional modules of *G. elata* co-expression network and further annotated their functions *via* gene set enrichment analysis.

### Orthologous Protein Prediction and Protein–Protein Interaction Network Construction

InParanoid (Sonnhammer and Ostlund, 2015) was a software developed by Perl script for constructing orthologous groups, and its normal operation could not do without the BLAST software. We used InParanoid software (Sonnhammer and Ostlund, 2015) to predict orthologous relationship between rice/maize and *G. elata* with a cutoff over 60% bootstrap. We then mapped the protein–protein interaction (PPI) network of maize and rice to *G. elata* to construct *G. elata* PPI networks.

### Gene Family Classification

We used the localized iTAK software to predict the transcription factors and transcription regulators of *G. elata* with default parameters, and the operation command was “iTAK.pl+protein_sequence.” We downloaded the hidden Markov model file of the conserved domain of ubiquitin proteases from the Ubiquitin and Ubiquitin-like Conjugation Database (UUCD) (Gao et al., 2013) and used the HMMER software to predict the ubiquitin proteases of *G. elata*. The *e*-value parameter used in this calculation process was derived from the threshold recommended by the UUCD (Gao et al., 2013). In order to predict EAR motif-containing proteins and CYP450 proteins, we first collected 20,542 EAR motif-containing proteins and 19,221 CYP450 protein sequences from the PlantEAR (Yang et al., 2018) and CYP450 databases (Nelson, 2009), respectively. Then, we predicted the orthologous relationship between collected proteins and *G. elata* proteins by InParanoid (bootstrap >60%) and further defined the EAR motif-containing proteins and CYP450 proteins based on the orthologous relationship.

### Search and Visualization Platform Construction

GelFAP was constructed based on CentOS Linux, Apache server, MySQL database, and PHP language. The software used for network visualization in the platform was a JavaScript package Cytoscape.js with open resources (Franz et al., 2016).

## Platform Contents

### Data Resources and Functional Annotation

**FIGURE 1:**
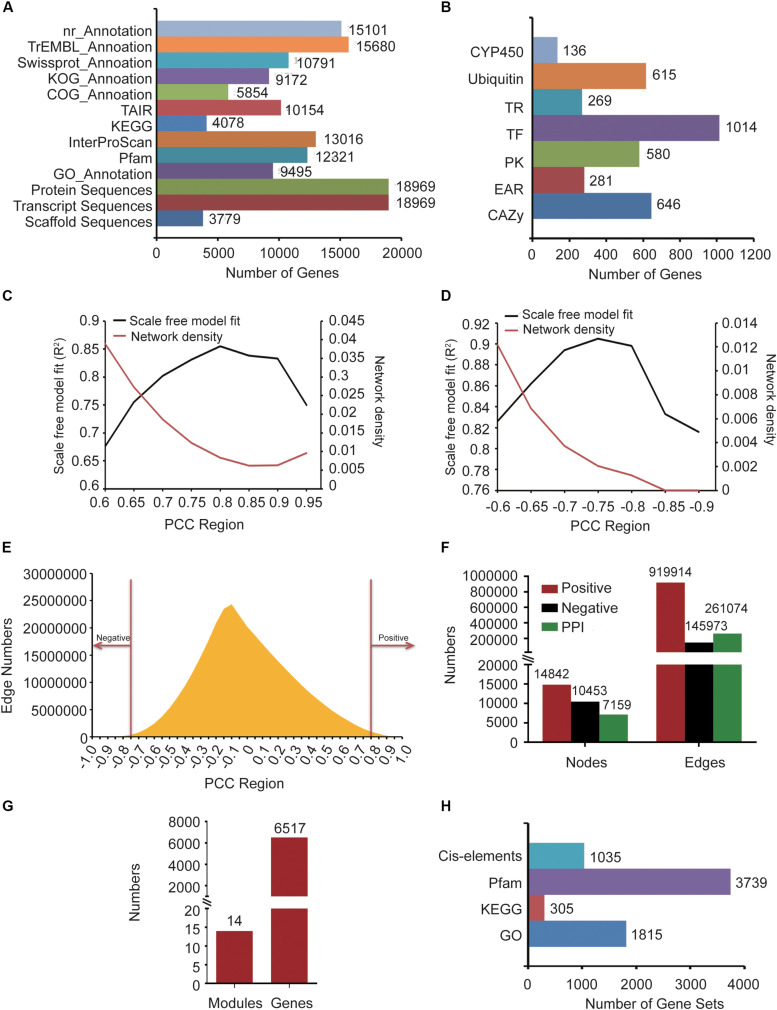
The information about *G. elata* gene function analysis platform. **(A)** Gene function annotation information. **(B)** Gene family classification information. **(C)** Network density and scale-free model fitting (*R*^2^) of the positive co-expression network based on changing Pearson correlation coefficient (PCC) cutoffs. **(D)** Network density and scale-free model fitting (*R*^2^) of the negative co-expression network based on changing PCC cutoffs. **(E)** Distribution diagram of the relationship between PCC and the number of edges. **(F)** Statistics of nodes and edges in the positive co-expression network, negative co-expression network, and PPI network. **(G)** Predicted gene functional modules and involved genes. **(H)** The background gene sets of the GSEA and motif enrichment analysis tools.

*Gastrodia elata* genomic data, including 3,779 scaffold sequences, gene location files, gene sequences, 18,969 transcript sequences, and 18,969 protein sequences, was derived from the National Genomics Data Center (NGDC) (Accession number: GWHAAEX00000000) of China produced by the National Resource Center for Chinese Materia Medica of China Academy of Chinese Medical (Yuan et al., 2018). The gene functions of *G. elata* were annotated by comparing nucleic acids or protein sequences with various functional annotation databases, including nr, KOG, TAIR (Reiser et al., 2017), COG, Swiss-Prot, and TrEMBL (Figure 1A). In addition, 27 transcriptome data samples were obtained from the SRA in NCBI (Accession number: SRP064423, SRP108465 and SRP118053) and 12 samples were produced by our group. We used the InterProScan (Jones et al., 2014) software to obtain GO terms of 9,495 genes and InterProScan domain annotations of 13,016 genes. The GO annotations were obtained from Gene Ontology Consortium (Gene Ontology Consortium, 2015). Pfam domain annotation information of 12,321 genes was predicted by the local PfamScan tool (El-Gebali et al., 2019). KEGG orthology annotation information of 4,078 genes was predicted by GhostKOALA (Kanehisa et al., 2016), which was supported by the KEGG website. Finally, the orthologous relationship between *G. elata* and *Arabidopsis thaliana* was analyzed by the InParanoid tool, and *Arabidopsis thaliana* annotation information of 10,154 genes in *G. elata* was obtained (Figure 1A).

### Gene Family Identification

Pfam is a protein family database, which contained multiple sequence alignment results and hidden Markov model (HMM) profiles of conserved regions from many gene families (El-Gebali et al., 2019). HMMER is a homolog searching tool based on HMM profiles (Potter et al., 2018). The gene families could be identified by combining Pfam with HMMER. Several platforms could also be used to identify gene families; for example, the analysis tools provided by the iTAK website were used for the identification of transcriptional regulators and protein kinases (Zheng et al., 2016), and HMM profiles offered by the UUCD database were used to identify members of the ubiquitin protease family (Gao et al., 2013). In addition, gene families could also be predicted by the orthologous relationship between different species.

To identify the CYP450 gene family numbers, 20,657 CYP450 protein sequences were downloaded from the CYP450 website (Nelson, 2009). Then, we constructed a library according to the downloaded CYP450 protein sequences and aligned the *G. elata* protein sequences with this library. From the results, we obtained 1,455 protein sequences whose *e*-value was less than 1e-5. Among them, 136 protein sequences with the CYP450 domain (PF00067.21) were identified as candidate members of the CYP450 family by HMMER. We used the iTAK software to identify the transcription factors, transcription regulators, and protein kinases of *G. elata* and obtained 1,014 transcription factors, 269 transcription regulators, and 580 protein kinases. We also used UUCD’s HMM profile to predict the ubiquitin proteases of G. elata, and 615 ubiquitin proteases were identified. To identify the carbohydrate-active enzymes (CAZy), we downloaded the genes of A. thaliana CAZy gene family from the CAZy database (Lombard et al., 2014), matched the CAZy gene family to *G. elata* according to their orthologous relationship, and predicted 646 CAZy genes of *G. elata* (Figure 1B). We also collected the EAR motif-containing proteins of 71 plants from the PlantEAR platform (Yang et al., 2018) and identified 281 EAR motif-containing proteins in *G. elata* according to their orthologous relationship (Figure 1B).

### Network Construction and Functional Module Identification

#### Co-expression Network

After removing the non-compliant transcriptome data samples by FastQC tools, we obtained 39 *G. elata* transcriptome data samples, including RNA-seq samples of SRP108465, SRP064423, SRP279888, and SRP118053 in SRA (Supplementary Table S1). The reads of RNA-seq samples were mapped to the *G. elata* genome and detailed alignment information was obtained by TopHat (Supplementary Table S1). In addition, the FPKM expression values of genes in each sample were obtained by computation using the Cufflinks software. Then, we calculated the PCC value between every two genes in different samples by WGCNA package of R language. Biological networks are usually scale-free networks and the network density is relatively low. Based on this principle, we analyzed PCC value over 0.6, 0.65, 0.7, 0.75, 0.8, 0.85, 0.9 and 0.95 to evaluate the scale-free model fitting index *R*^2^ and network density of the positive co-expression network. PCC > 0.8 had the largest scale-free model fitting index (*R*^2^) and the network density was relatively low (Figure 1C). We also chose the PCC threshold of the negative co-expression network based on the same method (Figure 1D). Finally, we chose PCC > 0.8 and PCC < −0.75 to determine the positive co-expression network and the negative co-expression network, respectively, (Figure 1E). We obtained a positive co-expression network with 14,842 nodes and 919,914 edges and a negative co-expression network with 10,453 nodes and 145,973 edges (Figure 1F).

#### Protein–Protein Interaction Network

The PPI network of maize and rice had been constructed in recent years (Zhu et al., 2016; Liu et al., 2017). So, we constructed the *G. elata* PPI network by predicting the orthologous relationship between maize and *G. elata* and mapped the maize PPI network to *G. elata*. By the same method, we also mapped the rice PPI network to *G. elata*. Finally, we obtained a PPI network with 7,159 nodes and 261,074 edges (Figure 1F).

#### Functional Module Identification

The co-expression network we constructed covered 14,842 genes, so we used the WGCNA to divide these genes into modules. WGCNA is a method used to construct a gene co-expression network based on gene expression profiles. By evaluating the relationship between soft threshold and scale-free model fitting index, we chose 7 as the soft threshold (Supplementary Figure S1A). Similarly, the relationship between soft threshold and mean connectivity showed that a soft threshold of 7 had a lower mean connectivity (Supplementary Figure S1B). Finally, we merged the modules after performing the dynamic tree cutting algorithm and then further identified gene functional modules based on the similarity between modules (Supplementary Figure S1C). We obtained 14 functional modules with 6,517 genes (Figure 1G).

#### Functional Enrichment Analysis Tools

We annotated *G. elata* genes by gene sets of 1,815 GO annotations, 305 KEGG orthology and 3,739 Pfam (Figure 1H). Then, we constructed the GSEA online tool by the algorithm described in the “Materials and Methods” section.

Motifs are short and conserved sequences of the gene promoter region. It could be recognized by various transcription factors and participated in the regulation of gene expression. We also collected 1,035 motifs from the PlantEAR (Yang et al., 2018) and ccNET platforms (Figure 1H; You et al., 2017). Using the motif analysis algorithm in the “Materials and Methods” section, we constructed an online motif enrichment analysis tool, which could perform motif analysis for the gene of *G. elata*.

#### The Structure of GelFAP

**FIGURE 2:**
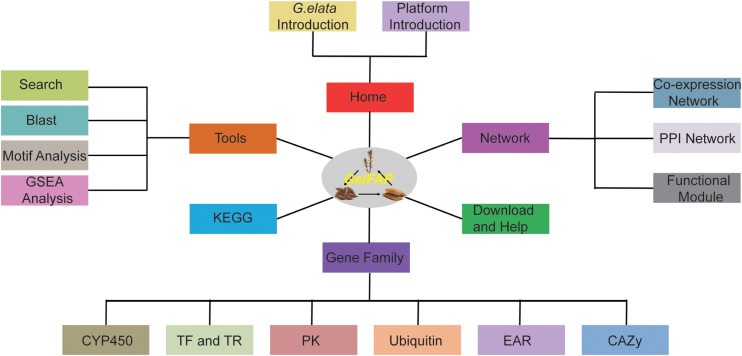
The framework structure of *G. elata* gene function analysis platform. GelFAP included six main sections. Home section was the introduction of *G. elata* and the platform. The network section contained the co-expression network, PPI network, and function modules. The gene family section contained CYP450 family genes, transcription factors, transcription regulators, protein kinases, ubiquitin proteasomes, CAZy genes, and EAR motif-containing proteins. Tools section included Search, BLAST, motif analysis, and GSEA toolkit. KEGG, Download and Help became section separately.

Based on the constructed gene co-expression networks, gene family classification, and functional analysis tools, the *G. elata* gene function analysis platform was constructed. The platform contained six main sections, namely Home, Browse, Gene family, Tools, KEGG, and Download and Help (Figure 2). Among them, there were network search and module search secondary menu functions under the network. The Tools section contained four secondary menus – Search, BLAST analysis, GSEA analysis, and cis-element analysis. The Gene family section contained CYP450, transcription factors, protein kinases, ubiquitin proteases, carbohydrate-active enzyme families, and EAR motif-containing proteins. The Pathway section contained pathways predicted by GhostKOALA (Kanehisa et al., 2016). In addition, the platform also provided the Download and Help page to assistant users to obtain data sources and help. The construction of the platform may contribute to the functional analysis of *G. elata* genes.

## Application

### Analysis of Putative Gastrodin Biosynthesis-Related Genes

The gastrodin biosynthesis may be regulated by CYP450, UGT, PAL, C4H, 4-HBS, and ADH family genes (Bai et al., 2016; Tsai et al., 2016). As shown in Supplementary Figure S2, many genes in this pathway had an obvious co-expression relationship. The PAL gene had a co-expression relationship with the C4H, CYP450, and ADH genes, and the CYP450 gene also had a co-expression relationship with UTG and ADH. Therefore, there may be an important synergistic relationship between them and they further participated in the regulation of gastrodin biosynthesis (Supplementary Figure S2).

A previous study had indicated that the CYP51G1 gene may be involved in the biosynthesis of gastrodin (Tsai et al., 2016), so we guessed that the function of this gene may be regulated by transcription factors that targeted on its upstream. We used the motif enrichment analysis tool to predict the transcription factors that might target on the CYP51G1 gene promoter region and found that multiple transcription factors were significantly enriched, including DRE1, MADS, and HD-zip transcription factors (Supplementary Figure S3). Therefore, these transcription factors may be the most probable genes that participated in the biosynthesis of gastrodin by regulating the CYP51G1 gene.

We selected the top 300 genes co-expressed with Arabidopsis CYP51G1 from the ATTED-II database (Obayashi et al., 2018) and compared them with the top 300 co-expressed genes of *G. elata* CYP51G1 (Supplementary Figure S4). The results demonstrated that there were 19 pairs of orthologous relationship. It had been reported that many genes of Arabidopsis had different functions (Supplementary Figure S4). For example, CPI1 (AT5G50375) was related to plant defense response (Cao et al., 2020), mMDH1 (AT1G53240) may be related to plant response to low temperature (Nakaminami et al., 2014), PGD1 (AT1G64190) could regulate the growth of Arabidopsis (Lim et al., 2009), TBL35 (AT5G01620) was related to xylan acetylation and growth (Yuan et al., 2016), and ARA12 (AT5G67360) was related to release mucilage of seed coat (Rautengarten et al., 2008). Therefore, these reported genes in Arabidopsis may help to predict the function of *G. elata* CYP51G1 gene.

### GAFP Identification and Functional Analysis

**FIGURE 3:**
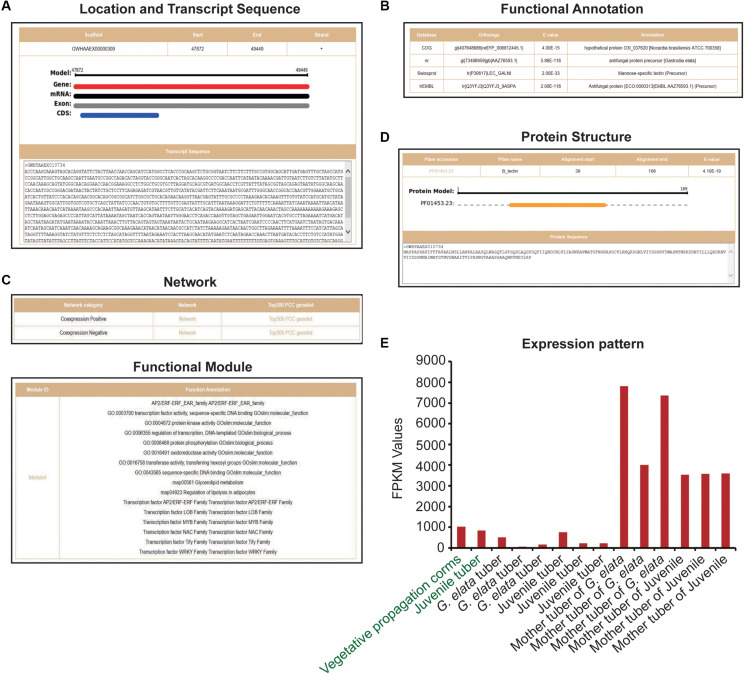
Gene details of *GAFP4*. **(A)** Location and transcript sequences. **(B)** Functional annotations. **(C)** Network and functional modules. **(D)** Protein structure. **(E)** Expression pattern of *GAFP4* gene; green represents transcriptome samples of SRP064423, and black represents transcriptome samples produced by us.

We obtained 12 *G. elata* mannose-binding lectin antifungal protein (GAFP) sequences from previous researches (Wang et al., 2016; Wang et al., 2019) and GenBank. By comparing these sequences with *G. elata* protein sequences, we obtained 23 protein sequences (*e*-value < 1e-3) and further identified them by the protein domain B_lection (PF01453.23). Finally, 19 *G. elata* proteins were identified as mannose-binding lectin antifungal proteins (Supplementary Table S2). The heterologous expression of *G. elata* GAFP4 gene (GWHGAAEX010734) in Arabidopsis thaliana could increase the resistance against *Botrytis cinerea*, and the heterologous expression of GAFP4 in cotton could also increase the resistance against Verticillium wilt (Wang et al., 2016; Wang et al., 2019). Here, we took *G. elata* antifungal protein GAFP4 as an example to analyze its functions by GelFAP. We searched the gene details and obtained the structure information and transcript sequences (Figure 3A), annotation information (Figure 3B), networks and functional modules (Figure 3C), protein structure and sequences (Figure 3D), and expression values (Figure 3E). We found that this gene had only one exon and CDS, and gene length was 1,557bp (Figure 3A). In addition, the functional annotation information indicated that this gene was annotated as an antifungal protein in the nr and TrEMBL databases (Figure 3B). Protein structure and sequence information suggested that this protein had a B_lectin domain. Related researches showed that the protein with B_lectin domains had antibacterial and antiviral functions (Cox et al., 2006; Sun et al., 2016; Wang et al., 2016; Wang et al., 2019; Yin et al., 2019; Figure 3D). Therefore, this domain may be an important structure for GAFP4 to perform its function.

**FIGURE 4:**
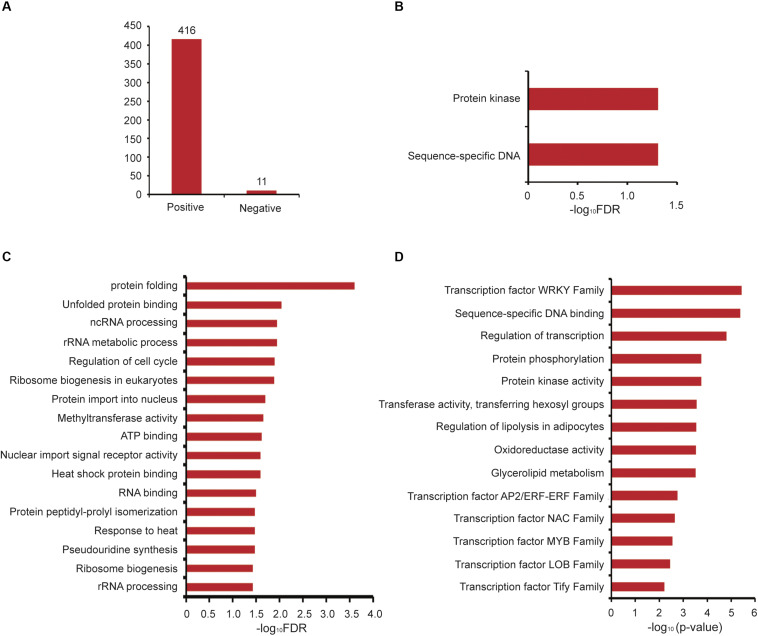
Co-expression and functional module analysis of *GAFP4*. **(A)** The number of positive and negative co-expressed genes of *GAFP4* gene. **(B)** GSEA of *GAFP4* positive co-expressed genes. **(C)** GSEA of *GAFP4* negative co-expressed genes. **(D)** GSEA annotation for the module that contained *GAFP4*.

Next, we analyzed GAFP4 gene function by the co-expression network, and the search results showed that GAFP4 had a positive co-expression relationship with 416 genes and a negative co-expression relationship with 11 genes (Figure 4A and Supplementary Table S3). GSEA of GAFP4 positive co-expressed genes revealed that this gene might have functions of protein kinase activity and sequence-specific DNA binding (Fisher’s exact test, FDR < 0.05) (Figure 4B). Therefore, GAFP4 may be co-expressed with several transcription factors (TFs) to perform its DNA-binding function. GSEA of GAFP4 negative co-expressed genes revealed its possible function in heat response, protein folding, methyltransferase, regulation of cell cycle, and so on (Fisher’s exact test, FDR < 0.05) (Figure 4C). In addition, we obtained a function module that contained GAFP4 (Supplementary Table S4). GSEA of this module showed significant enriched transcription factor family members, including ERF, MYB, and WRKY families. Moreover, protein kinase activity, transferase activity, oxidoreductase activity, and glycerolipid metabolism were also enriched in the module (Fisher’s exact test, *P* value < 0.05) (Figure 4D). When plants were infected by bacteria or viruses, the plant transcription factor families ERF (Wang et al., 2018; Zhu et al., 2019), MYB (Ibraheem et al., 2015; Shan et al., 2016), WRKY (Chen et al., 2013; Peng et al., 2016; Wang et al., 2017; Gao et al., 2018; Liu et al., 2018; Li et al., 2020), and protein kinase (Kim and Hwang, 2011; Shen et al., 2012) showed response functions. Therefore, GAFP4 may be co-expressed with many antibacterial TFs or form functional modules with TFs to further play its role in antibacterial defense response. Therefore, by analysis of GAFP4 gene in GelFAP, we found that it might have antibacterial effect functions. At present, its antibacterial function has been verified in cotton and Arabidopsis (Wang et al., 2016; Wang et al., 2019), and many other functions still need to be explored in the future.

## Discussion

*Gastrodia elata* is a valuable traditional Chinese herbal medicine and has numerous important pharmacological roles. The whole genome sequencing of *G. elata* has been completed in recent years and its transcriptome data also has a certain accumulation (Tsai et al., 2016; Yuan et al., 2018). In this study, we firstly used the genome and transcriptomes of *G. elata* to construct *G. elata* gene co-expression networks and functional modules and provided related gene function analysis and annotation tools, including the BLAST search tool, GSEA tool, and motif enrichment analysis tool. The gene co-expression networks were of great significance for exploring gene functions, such as comparing networks between orthologous gene pairs in model specie and *G. elata*, which could provide more information for gene function researches. Similarly, gene function enrichment analysis tools also played important roles in *G. elata* gene functional researches. For example, gene enrichment analysis tools could analyze possible downstream functions of differentially expressed genes in the transcriptome. Finally, the gene families such as CYP450, transcription factors, protein kinases, ubiquitin proteases, and carbohydrate-active enzymes were classified and predicted, and the results were integrated into the *G. elata* gene functional analysis platform. Therefore, our platform can provide more data sources and analysis methods for researchers to study the gene function of *G. elata*, which may improve the efficiency of the research for *G. elata* genes.

*Gastrodia elata* established a symbiotic relationship with Armillaria during the growth process, and it was reported that GAFPs played important roles in establishing this relationship, but which kind of GAFPs were not mentioned. We identified 19 *G. elata* GAFPs based on the sequence information provided by the platform, and provided candidate genes for the follow-up research on the establishment of symbiotic relationship. Furthermore, we took the *GAFP4* gene as an example to introduce the application method of the platform. The analysis results indicated that *GAFP4* might be involved in various regulatory processes including antibacterial, and it had also been reported to have the function of antibacterial (Wang et al., 2016; Wang et al., 2019). Therefore, the platform we built has a certain feasibility and practicality.

The *G. elata* gene function analysis platform is established by us for the first time. Users can submit their interesting genes to the platform and then obtain information of various existing and processed annotations. However, there is still much room for improvement in the accumulation of omics data. In the future, we will continue to update and maintain the *G. elata* gene function analysis platform, such as collecting and integrating more transcriptome, proteome, metabolome data, etc. We expect that this platform will contribute to the study of molecular mechanisms in the process of gastrodin biosynthesis, and further help to solve the problems about variety and quality improvement of *G. elata*.

## Data Availability Statement

Publicly available datasets were analyzed in this study. These data can be found here: https://trace.ncbi.nlm.nih.gov/Traces/sra/?study=SRP064423, https://trace.ncbi.nlm.nih.gov/Traces/sra/?study=SRP108465, https://trace.ncbi.nlm.nih.gov/Traces/sra/?study=SRP118053, and https://trace.ncbi.nlm.nih.gov/Traces/sra/?study=SRP279888.

## Author Contributions

JY designed this study. JY and QX constructed the platform and completed the draft. LD, LG, and JX produced and processed the transcriptome. ZS, WX, and YL participated in the construction of this platform. SY and QP participated in the revision of the manuscript. TZ, WJ, and LH directed this work and provided financial support.

## Conflict of Interest

The authors declare that the research was conducted in the absence of any commercial or financial relationships that could be construed as a potential conflict of interest.
