# Supplementary material for: GelFAP: Gene Functional Analysis Platform for Gastrodia elata
Source: Front Plant Sci. 2020 Oct 22;11:563237. doi: 10.3389/fpls.2020.563237 (PMC7642037; doi:10.3389/fpls.2020.563237)
Supplement: Supplementary file 2 [file Table_2.DOCX]

Supplementary Material

**GelFAP: Gene functional analysis platform for *Gastrodia elata***

Jiaotong Yang^1#^*, Qiaoqiao Xiao^1#^, Jiao Xu^1^, Lingling Da^2^, Lanping Guo^3^, Yue Liu^4^, Wenying Xu^2^, Zhen Su^2^, Shiping Yang^2^, Qi Pan^1^ and Tao Zhou^1^*

^1^Guizhou University of Traditional Chinese Medicine, Guizhou 550025, China

^2^College of Biological Sciences, China Agricultural University, Beijing 100193, China

^3^National Resource Center for Chinese Meteria Medica, Chinese Academy of Chinese Medical Sciences, Beijing 100700, China.

^4^College of Horticulture, Qingdao Agricultural University, Qingdao, 266109, China.

***To whom correspondence should be addressed.**

**Jiaotong Yang (**y_jiaotong@163.com**); Tao Zhou (**taozhou88@163.com**)**

# 1 Supplementary Figures and Tables

## Supplementary Tables

**Table S1**. Summary of RNA-seq datasets collected in *G. elata*.

**Table S2**. *G. elata* mannose-binding lectin antifungal proteins (GAFPs).

**Table S3**. Co-expressed genes of *GAFP4*.

**Table S4**. Function module that contained GAFP4..

## Supplementary Figures

**
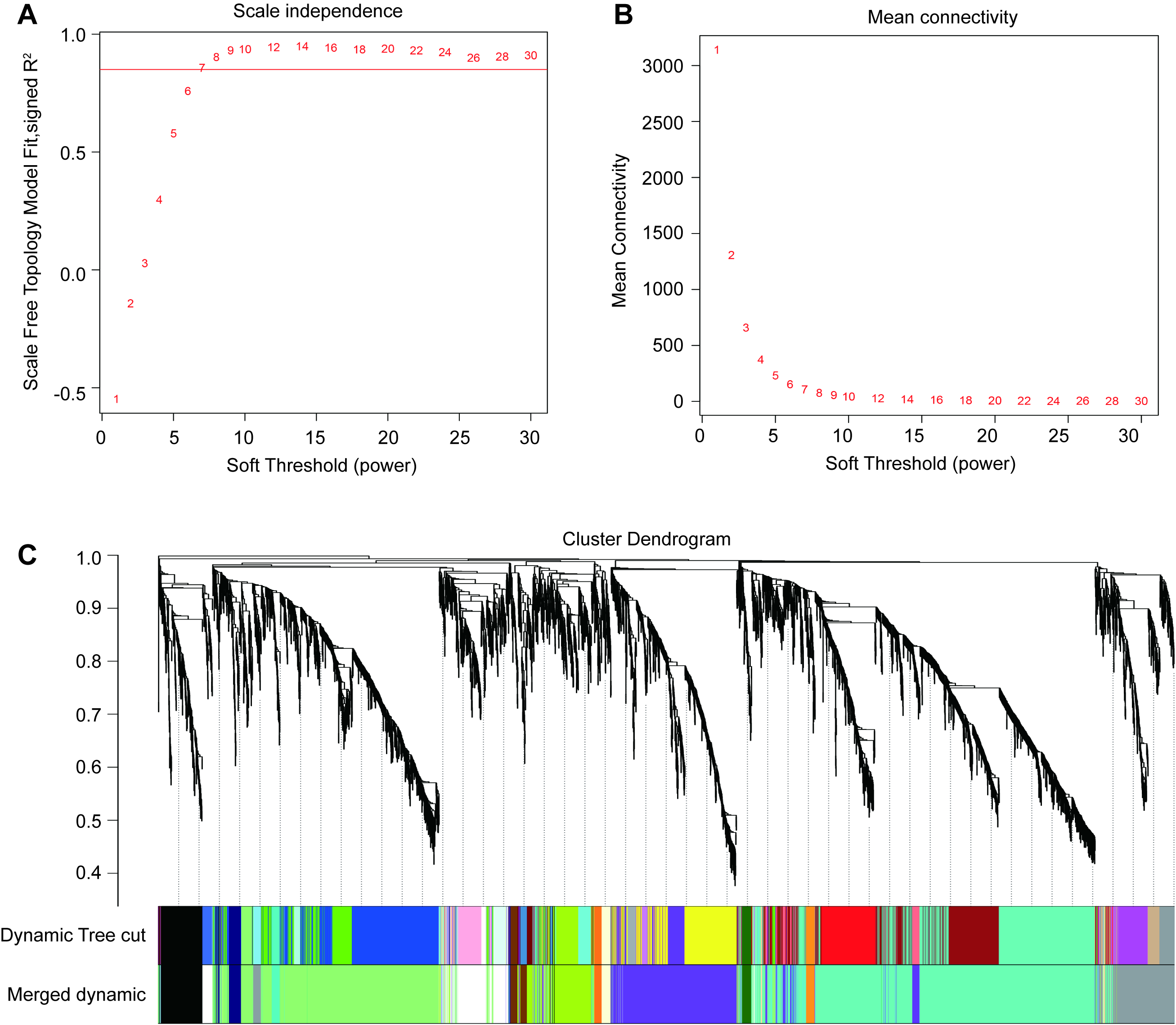
**

**Figure S1.** Selection of the soft threshold and the functional module identification. (A) The distribution relationship between soft threshold and scale free model fitting index R^2^. (2) The distribution relationship between soft threshold and mean connectivity. (C) Modules display after dynamic tree cutting and merging.


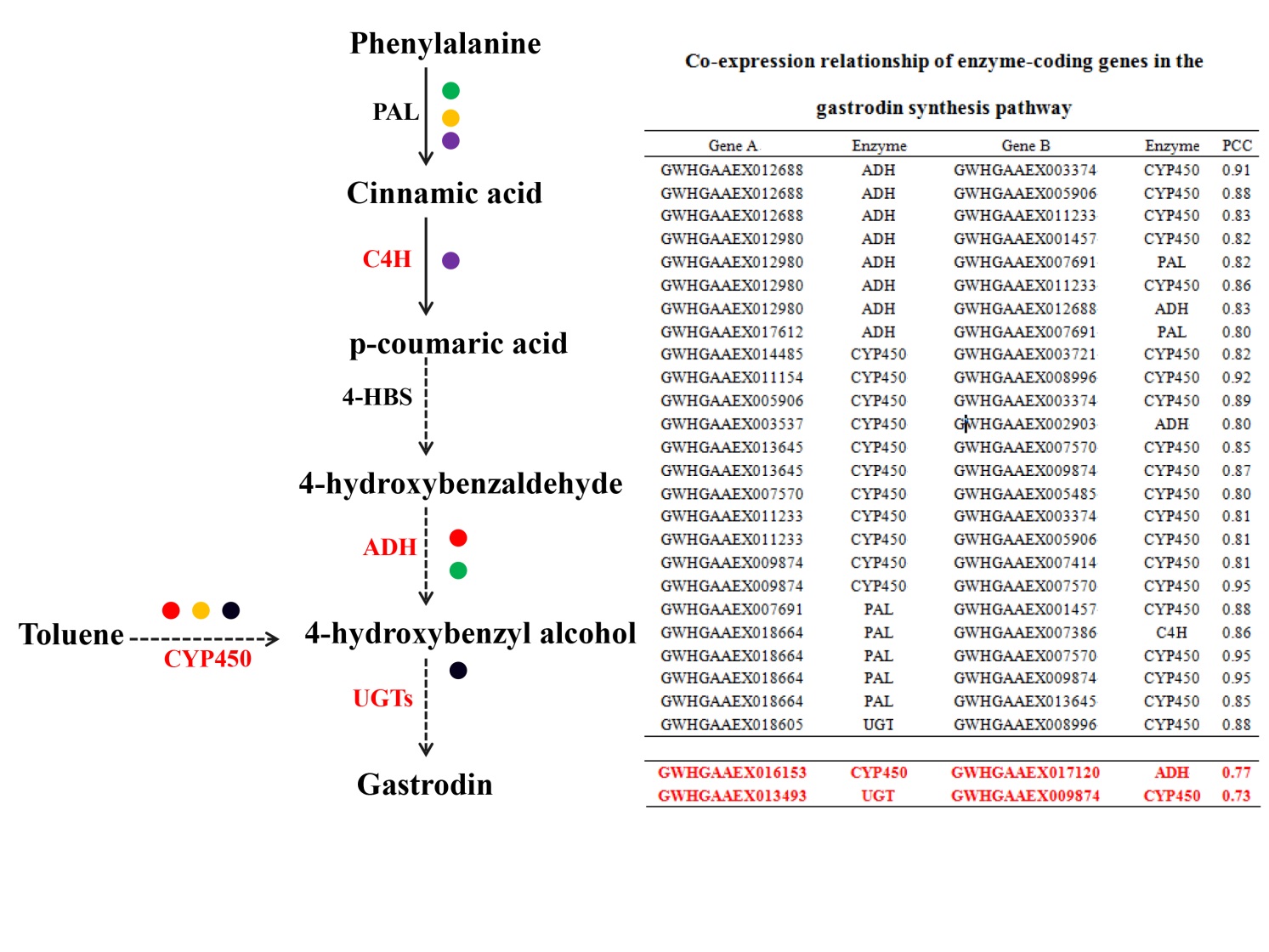
**Figure S2.** Co-expression analysis of key enzyme genes in the possible gastrodin synthesis pathway. For the left figure, the dots in different reaction with the same colour mean that the key enzymes have co-expression relationship. The right figure is the detailed co-expression relationship of key enzyme genes.


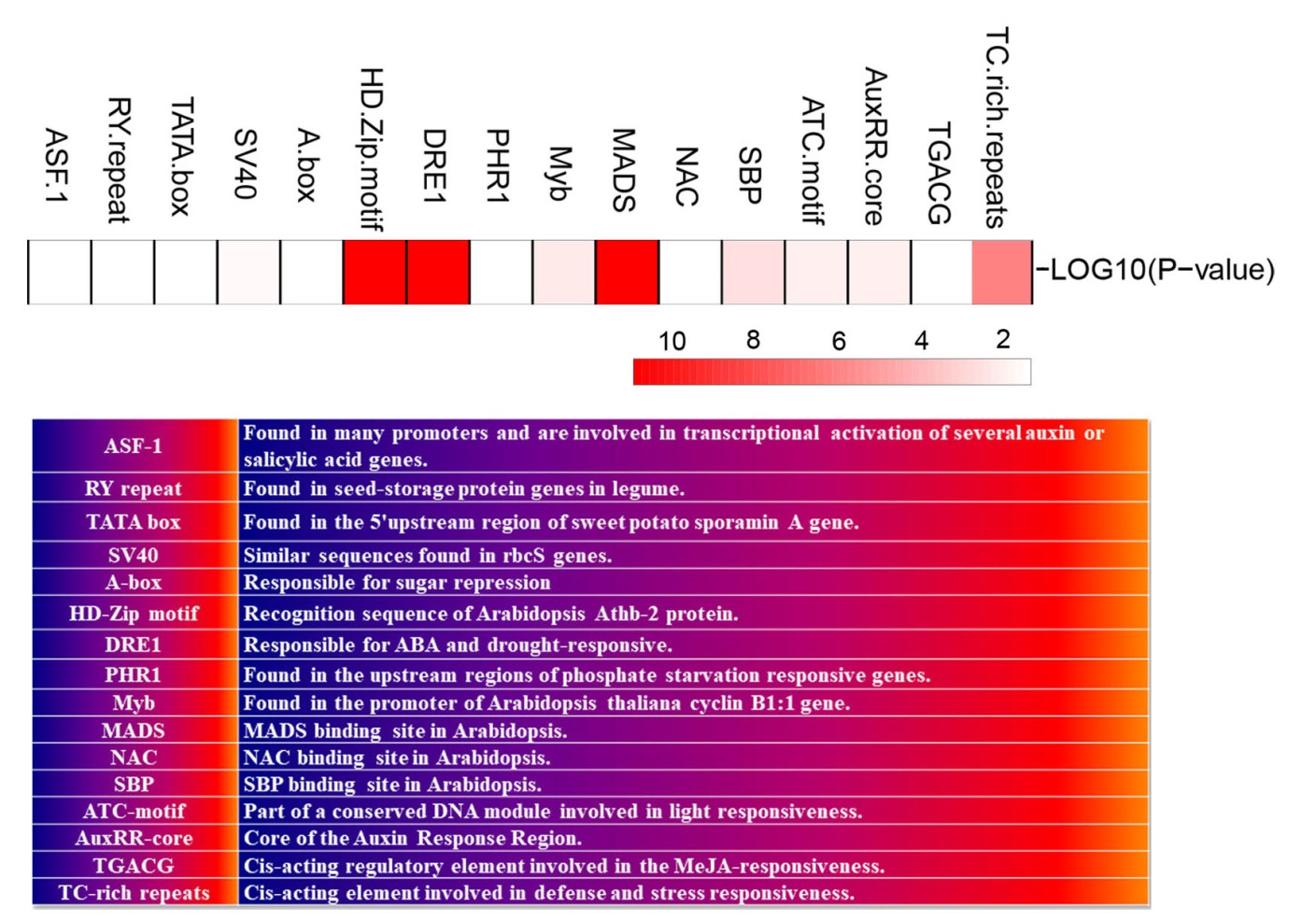


**Figure S3.** The results of motif enrichment analysis on the promoter region of *CYP51G1* gene by *G. elata* gene functional analysis platform.

**
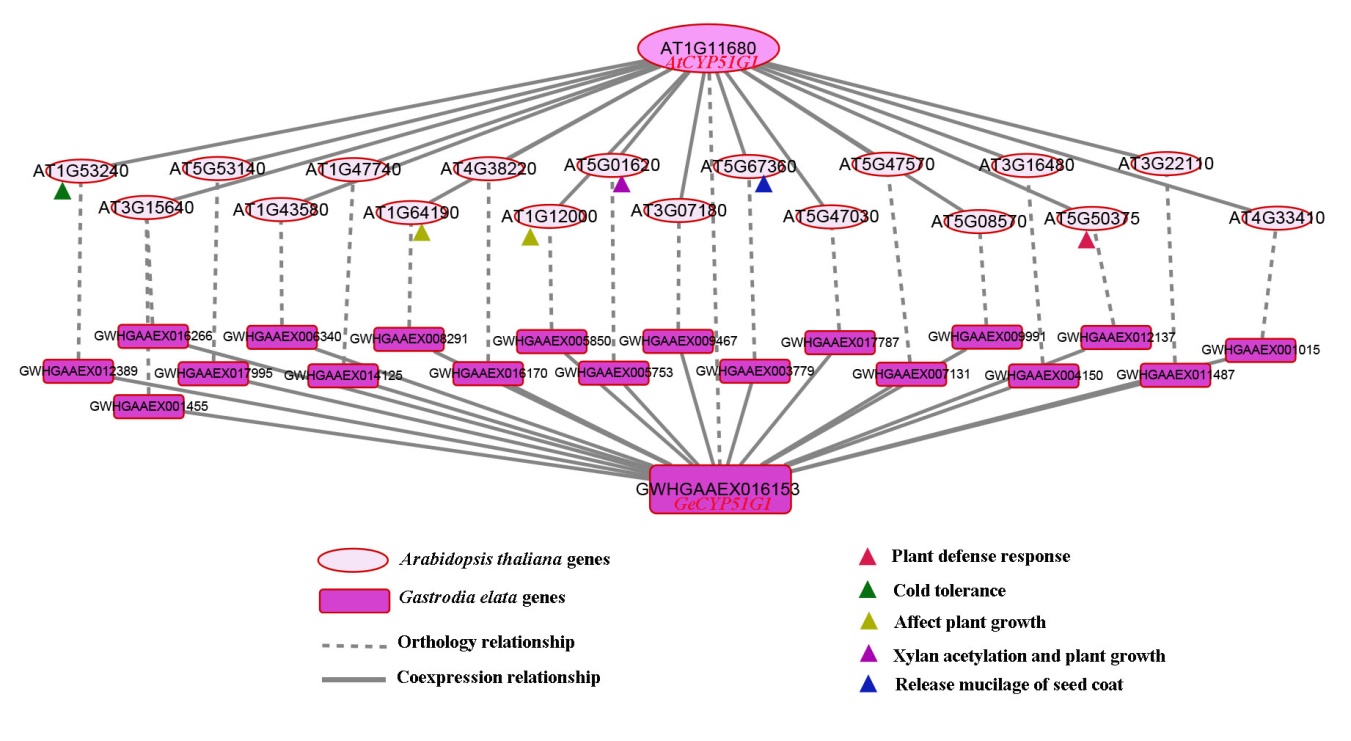
**

**Figure S4.** The results of top 300 co-expression network comparison between *Arabidopsis thaliana* and *Gastrodia elata*. The ellipse represents the *Arabidopsis thaliana* genes, the rectangle represents the *Gastrodia elata* genes, the dotted line represents the orthologous relationship, the solid line represents the co-expression relationship, and the triangles with different colours represent the reported functions of the *Arabidopsis thaliana* genes.
